# FDG-PET/CT based small volume accelerated immuno chemoradiotherapy in locally advanced NSCLC (PACCELIO) – a randomized, open-label, multicenter phase II trial protocol

**DOI:** 10.1186/s12885-026-15901-x

**Published:** 2026-04-17

**Authors:** Rami El Shafie, Jonas Willmann, Eleni Gkika, Tanja Schimek-Jasch, Matthias Miederer, Farastuk Bozorgmehr, Frank Griesinger, Farkhad Manapov, Petros Christopoulos, Thomas Hehr, Markus Hecht, Rebecca Bütof, Martin Stuschke, Matthias Guckenberger, Gerald Schmid-Bindert, Jan Meiners, Sascha Herzer, Sonja Hartmann, Joana Lamché, Stefan Rieken, Ursula Nestle

**Affiliations:** 1 Department of Radiation Oncology, University Hospital Göttingen, University Medical Center Göttingen (UMG), Robert-Koch-Str. 40, Göttingen, 37075 Germany; 2 Department of Radiation Oncology, University Hospital Zurich, University of Zurich, Zurich, Switzerland; 3 Department of Radiation Oncology, University Hospital Bonn, Bonn, Germany; 4 Department of Radiology, Medical Center, Faculty of Medicine, University of Freiburg, Freiburg, Germany; 5 Department of Translational Imaging in Oncology, National Center for Tumor Diseases (NCT/UCC) Dresden: Faculty of Medicine and University Hospital Carl Gustav Carus, University of Technology Dresden (TUD), German Cancer Research Center (DKFZ) Heidelberg, and Helmholtz-Zentrum Dresden-Rossendorf (HZDR), Dresden, Germany; 6 National Center for Tumor Diseases (NCT), Heidelberg, Germany; 7 Department of Thoracic Oncology, Thoraxklinik, Heidelberg University Hospital and National Center for Tumor Diseases (NCT), NCT Heidelberg, a partnership between DKFZ and Heidelberg University Hospital, Heidelberg, Germany; 8 Translational Lung Research Centre Heidelberg (TLRC-H), German Centre for Lung Research (DZL), Heidelberg, Germany; 9 Department of Internal Medicine Oncology, Pius Hospital, University Medicine, Oldenburg, Germany; 10 Department of Radiation Oncology, University Hospital of Munich (LMU), Munich, Germany; 11 BU Oncology – Medical Affairs, AstraZeneca GmbH, Hamburg, Germany; 12 Clinical Operations – Medical Writing, Alcedis GmbH, Gießen, Germany; 13 Department of Radiation Oncology, Kliniken Maria Hilf, Moenchengladbach, Germany; 14 V. Medical Clinic, University Medical Center Mannheim, Heidelberg University, Mannheim, Germany; 15 Department of Radiation Oncology, Marienhospital Stuttgart, Stuttgart, Germany; 16 Department of Radiation Oncology, Saarland University Medical Center, Homburg, Germany; 17 Department of Radiotherapy and Radiation Oncology, Faculty of Medicine and University Hospital Carl Gustav Carus, TUD Dresden University of Technology, Dresden, Germany; 18 Department of Radiation Therapy, University Hospital Essen, Essen, Germany

**Keywords:** Radiotherapy, Hypofractionation, NSCLC, Immunotherapy, Radoitherapy

## Abstract

**Background:**

Standard treatment for unresectable stage III non-small cell lung cancer (NSCLC) involves chemoradiation (CRT) followed by PD-L1 targeting immune checkpoint inhibition (IO). Integrating 18 F-fluorodeoxyglucose-positron emission tomography (FDG-PET)/CT into radiotherapy planning reduces toxicity, improves CRT outcomes, and potentially enhances immunotherapy response. Hypofractionated, accelerated CRT shortens treatment time, improves compliance, and may increase CRT completion rates, qualifying more patients for consolidative IO.

**Methods/design:**

PACCELIO is a multinational, multicenter, randomized, phase II trial comparing the safety and efficacy of FDG-PET/CT based, volume-reduced, hypofractionated CRT with consolidation durvalumab to standard-volume, conventionally fractionated CRT with consolidation durvalumab in inoperable stage III NSCLC. One hundred and ten patients will be enrolled in Germany, Austria, and Switzerland, randomized 1:1, stratified by age, NSCLC stage, and site. The primary objective is to increase completion of CRT with successful transition to consolidation immunotherapy. Secondary outcomes include locoregional and distant tumor control rates, overall survival, safety, and health-related quality of life (HR-QoL). Exploratory outcomes involve prognostic immunomarkers and PET parameters. The primary endpoint of treatment completion will be analyzed using Fisher's Exact test, with safety and efficacy outcomes analyzed via Kaplan-Meier.

**Discussion:**

The PACCELIO trial addresses a clinical need in stage III NSCLC by combining FDG-PET/CT based RT volume reduction with hypofractionated accelerated CRT and durvalumab immunotherapy. This approach aims to improve treatment completion, compliance, tumor control, and reduce toxicity. The trial will provide insights into the efficacy and safety of this combined treatment, potentially influencing future standards in stage III NSCLC treatment. Recruitment began in July 2024, with ongoing enrollment.

**Trial registration:**

Clinicaltrials.gov identifier: NCT06102057 (Date of initial registration: 26th October 2023), https://clinicaltrials.gov/study/NCT06102057.

**Supplementary Information:**

The online version contains supplementary material available at 10.1186/s12885-026-15901-x.

## Background

### Treatment strategies for stage III non-small cell lung cancer

Lung cancer is a prevalent disease with significant morbidity and mortality rates, and a leading cause of cancer deaths worldwide.

Globally, more than 2.2 million new cases of lung cancer were diagnosed in 2020. At more than 1.7 million deaths annually, lung cancer was the leading cause of cancer-related mortality worldwide in 2020 [1]. Non-small cell lung cancer (NSCLC) is the most common form of lung cancer, and accounts for about 80–90% of all cases [2]. About 25% of these patients present with locally advanced (i.e. stage III) disease at first diagnosis. Despite advances in treatment effectiveness and reduced perioperative morbidities, surgery is viable for only a minority of patients with stage III NSCLC. The recommended treatment for unresectable locally advanced (stage III) NSCLC is concurrent or sequential chemoradiation (CRT) followed by consolidation immunotherapy with durvalumab, as per the PACIFIC trial [3–6]. 

For inoperable NSCLC, radiotherapy is typically fractionated to minimize organ toxicity while maximizing tumor control [7]. Radiosensitizing chemotherapy, predominantly cisplatin-based, enhances the effectiveness of RT and is delivered simultaneously or sequentially in patients not eligible for simultaneous CRT [8]. Advances of immune checkpoint inhibition, with monoclonal antibodies targeting PD-1 or PD-L1, such as pembrolizumab, nivolumab, or durvalumab, have significantly improved outcomes of various cancers, including NSCLC. Durvalumab, specifically, has been established as the standard of care consolidation therapy for locally advanced NSCLC patients without disease progression after CRT, based on improved tumor control and survival rates shown in the PACIFIC trial [3–5]. 

Early post-PACIFIC real-world series reported that up to 50% of patients did not proceed to consolidation durvalumab after CRT due to progression, PD-L1 expression status, or toxicity [9]. With accumulated experience and streamlined pathways, contemporary cohorts show higher transition rates (≈ 60–68%) [10–14]. Nevertheless, about 45% of patients will progress or die within one year after the start of consolidation immunotherapy with durvalumab, underscoring the need to minimize CRT-related attrition and enable timely consolidation [15]. Strategies to reduce toxicity, to predict response rates and adapt treatment schedules accordingly to improve outcomes are urgently needed for these patients. Furthermore, systemic inflammation, indicated by biomarkers like the neutrophil-lymphocyte ratio (NLR) and the advanced lung cancer inflammation index (ALI), correlates with poorer prognosis and inferior response to immunotherapy in NSCLC patients [16, 17]. Studies have shown that baseline and post-treatment NLR values can predict overall survival and treatment response. Similarly, ALI - encompassing NLR, body mass index (BMI), and serum albumin levels - has emerged as a predictive marker in advanced NSCLC [18, 19]. These biomarkers, easily derived from peripheral blood samples, could play an important role in outcome prediction and clinical decision making. However, they lack validation in prospective, longitudinal studies, especially in stage III NSCLC treated with CRT followed by consolidation immunotherapy.

### Integration of FDG-PET/CT for radiotherapy target volume definition

The integration of 18 F-fluorodeoxyglucose-positron emission tomography (FDG-PET)/CT for target volume definition marked a significant advancement in radiotherapy treatment planning for NSCLC patients. Traditionally, radiotherapy plans were largely based on 3-dimensional CT scans, typically with 2–3 mm slice thicknesses, often enhanced with contrast agents for better visualization of primary tumors and mediastinal lymph nodes. The integration of FDG-PET/CT into this process allows for a more accurate delineation of the gross tumor volume (GTV), and may therefore improve local tumor control while maintaining isotoxicity, as demonstrated in the PET-Plan trial [20, 21]. 

This precision in delineating the macroscopically visible GTV - including both the primary tumor and involved lymph nodes - is crucial for determining the clinical target volume (CTV), which also accounts for adjacent areas of microscopic disease spread. The use of FDG-PET/CT allows for a more nuanced approach to defining these volumes [22]. Historically, the inclusion of entire mediastinal lymph node levels was common, even if only partly affected. Additionally, elective levels were included without radiographic evidence of tumor, based on predictive models of locoregional spread. However, FDG-PET/CT imaging offers the potential to more accurately define the CTV by focusing on biologically active areas, reducing the need for overly broad margins that encompass elective, uninvolved lymph node levels [20, 23]. This concept has been prospectively tested in the international PET-Plan trial [24], with isotoxic dose-escalation either on conventional versus FDG-PET based target volumes. In the experimental arm, targeting the primary tumor and FDG positive mediastinal lymph node stations only, the locoregional control was improved significantly without an increased risk of isolated out-field recurrences. However, overall survival was not affected by this strategy.

### Potential benefits of hypofractionated, accelerated radiotherapy

Conventionally, radiotherapy is administered in 1.8–2 Gy doses per daily fraction, to a total dose of 60–66 Gy. Despite technological advancements, clinical trials have not supported dose escalation, primarily due to increased toxicity rates obviating the potential survival benefit from improved local tumor control [25, 26]. The toxicity rates are mostly related to key thoracic organs at risk such as the heart, lungs, and esophagus [26–28]. In the PET-Plan cohort, for example, there was a measurable correlation of the radiotherapy dose applied to the base of the heart with survival [29]. Furthermore, patients’ survival correlated with the quality of radiotherapy plans with respect to normal tissue protection [30]. 

Hypofractionated schedules have been explored, showing feasibility at comparable or even lower toxicity rates, but are not yet widely adopted. Hypofractionation, delivering single doses exceeding 2.2 Gy, aims to provide an accelerated radiotherapy regimen at comparable or even slightly escalated biologically effective dose (BED) [31–33]. This method not only addresses the radiation dose-response relationship for NSCLC but also aligns with the practical goal of reducing the duration of CRT. Shorter CRT treatment duration is particularly advantageous in the context of multimodality treatment of stage III NSCLC, as it may improve patient compliance and increase the likelihood of patients successfully proceeding to subsequent consolidation immunotherapy after CRT [34]. 

Improving eligibility for and increasing successful transition to consolidation immunotherapy after CRT is an unmet clinical need for patients with unresectable stage III NSCLC: More than half of potentially eligible patients do not receive consolidation immunotherapy due to incomplete or prematurely terminated CRT, and about a third do not complete durvalumab consolidation, often due to early progression or toxicities such as pneumonitis [11, 35]. The lung volume receiving radiation is closely correlated to the rate of pneumonitis, as confirmed in a recent study [36]. 

Taken together, these findings suggest that improving the spatial precision and reducing the treatment time of thoracic radiotherapy for stage III NSCLC could reduce toxicity and improve CRT completion rates enabling successful transition to consolidation immunotherapy with durvalumab.

### Research hypothesis

The PACCELIO randomized phase 2 trial is designed to establish feasibility and safety of a regimen combining FDG-PET/CT–guided, volume-reduced hypofractionated CRT with consolidating durvalumab immunotherapy.

The objectives of this trial are to: (i) improve feasibility in terms of an increased CRT treatment completion rate as compared to standard-volume conventionally fractionated CRT with consolidating immunotherapy, (ii) improve disease control and survival rates by reducing toxicity and facilitating immune-effects, and (iii) evaluate the role of immunomarkers derived from peripheral blood and quantitative imaging markers in predicting treatment outcome and prognosis.

## Methods/design

### Study design, setting, objectives, and characteristics of participants

The PACCELIO trial is a multinational, randomized, controlled, open-label, multicenter phase II trial to examine the feasibility and efficacy of FDG-PET/CT based, volume-reduced, hypofractionated CRT with consolidation durvalumab treatment (Experimental Arm) in unresectable stage III NSCLC. The primary objective is to increase the rate of CRT treatment completion and successful transition to concolidation immunotherapy, as compared to standard-volume, conventionally fractionated CRT with consolidation durvalumab treatment (Conventional Arm). Secondary outcomes include the rate of treatment-related adverse events (TRAEs) according to National Cancer Institute Common Terminology Criteria for Adverse Events (NCI-CTCAE) v5.0, as well as local, locoregional and distant tumor control rates, disease control rate and overall survival. Furthermore, health-related quality of life (HR-QoL) will be assessed according to EORTC QLQ-C30 and QLQ-LC13 questionnaires. Exploratory outcomes include the translational evaluation of prognostic immunomarkers, as well as radiotherapy planning and PET-specific imaging parameters.

In total, 110 patients are planned to be included in 12 sites (academic hospitals and associated centers) across Germany, Austria and Switzerland within 24 months. A complete list of participating sites will be regularly updated at Clinicaltrials.gov (NCT06102057). Eligible patients will be pre-screened in the multidisciplinary tumor board and invited to participate. At the time of inclusion, all patients must have histologically or cytologically confirmed NSCLC and present with locally advanced, unresectable (stage III) disease. All patients also need to have received a diagnostic FDG-PET/CT as part of standard of care (SOC) staging and for radiotherapy planning within a maximum of 21 days before the initiation of CRT. The main eligibility criteria are specified in Table 1.

**Table 1:** Main eligibility criteria for the PACCELIO trial

Inclusion Criteria	Exclusion Criteria
1. Written informed consent	1. Mixed small cell and NSCLC histology
2. Patients irrespective of sex and gender, aged 18 years or older at the time of signing the ICF	2. Neuroendocrine tumor
3. Patients with histologically or cytologically documented NSCLC who present with locally advanced, unresectable (stage III) disease	3. Distant metastases
4. Fit for simultaneous chemoradiotherapy and consolidation immunotherapy according to interdisciplinary consensus	4. Malignant pleural effusion or pericardial effusion
5. PD-L1-expression of ≥ 1% (tumor proportion score; TPS)	5. Acute superior vena cava obstruction
6. ECOG PS 0–1	6. Prior or current cancer treatment for NSCLC. Exception: Prior surgical resection of limited metachronous NSCLC (i.e., stage I or II) is permitted.
7. Tumor assessment by FDG-PET/CT within 21 days prior to start of chemoradiotherapy	7. Receipt of live attenuated vaccine within 30 days prior to the start of therapy.
8. Adequate pulmonary function test results o FEV1 > 40% AND o DLCO > 30%	8. Major surgical procedure within 28 days prior start of treatment.
9. Adequate bone marrow and organ function at enrolment	9. Prior exposure to immune-mediated therapy, including but not limited to, other anti- CTLA-4, anti-PD-1, anti-PD-L1 (including durvalumab), and anti-PD-L2 antibodies, including therapeutic anticancer vaccines
10. Adequate blood laboratory results as specified in study protocol	10. Current use of ongoing long-term immunosuppressive medication
11. Body weight of > 30 kg at enrolment	11. History of allogeneic organ transplantation
	12. Active or prior documented autoimmune or inflammatory disorders
	13. Uncontrolled intercurrent illness
	14. Oxygen dependence
	15. Acute inflammation of mediastinal lymph nodes/mediastinal lymphadenopathy
	16. History of another primary malignancy
	17. Active infection including tuberculosis, hepatitis B, hepatitis C, or human immunodeficiency virus (HIV)

*Abbreviations:* *ICF* Informed consent form, *NSCLC* Non-small cell lung cancer, *ECOG* *PS* Eastern Cooperative Oncology Group Performance Status, *FDG-PET/CT* 18F-fluorodeoxyglucose-positron emission tomography / computed tomography, *FEV1* Forced expiratory volume in 1 s, *DLCO* Diffusing capacity for carbon monoxide

### Study procedures

#### Enrollment and randomization

Site investigators will obtain written informed consent from all study participants prior to enrolling them into the study. Randomized allocation to the treatment arms will be done in a 1:1 fashion directly within the electronic case report form (eCRF). Randomization will be stratified by age (< 65 vs. ≥65 years), NSCLC stage (IIIA vs. IIIB/C) and study site. Specifically, stratification by site uses hierarchical biased-coin minimization (Signorini) with study site (defined as the participating treating institution) as a stratum, such that within each site any arm imbalance is corrected by assigning the next patient to the under-represented arm with 80:20 preference, reverting to 1:1 allocation when both site-specific and overall counts are balanced. No blinding is planned for this study; all treatments (CRT as well as durvalumab consolidation) are administered open-label.

#### Study treatment and follow-up

In the Experimental Arm, patients will receive FDG-PET-based target volume-reduced, hypofractionated, accelerated CRT at a total dose of 60.5–66 Gy in single doses of 2.75 Gy per daily fraction, prescribed to the PTV of the primary tumor and the GTV of bulky lymph nodes (≥ 3 cm in any diameter). The remainder of the PTV (with a locally expanded nodal CTV delineated according ACROP guideline, Option 2) will receive 53,9–58,8 Gy in single doses of 2,45 Gy. In the Conventional Arm, patients will receive conventionally fractionated CRT at a total dose of 60–66 Gy, prescribed homogeneously to the whole PTV (including a larger nodal CTV anatomically including the whole affected nodal stations according to the ACROP guideline Option 1).

Chemotherapy (both arms): Centers will administer an EMA-approved platinum doublet per institutional standard of care (cisplatin or carboplatin in combination with etoposide, vinorelbine, paclitaxel/docetaxel, or pemetrexed; gemcitabine is not permitted). Chemotherapy is given concurrently with radiotherapy; the first platinum dose is scheduled within ± 7 days of the first RT fraction. At least two cycles are planned, and two platinum doses must occur during the RT course. To achieve this in the hypofractionated arm, investigators are free to adapt chemotherapy within the scope of evidence-based regimens and institutional practice, e.g. in the context of past hypofractionation trials (e.g. SOCCAR, EORTC 08972–22973, CALGB 31102 [37–39]. Strategies may entail condensing conventional schedules so that two cycles fit within the 4-week RT course or use weekly/bi-weekly platinum regimens (e.g., platinum–taxane) or reducing concurrent chemotherapy to radiosensitizing doses (e.g., low-dose daily regimens). Chemotherapy is not continued beyond the end of RT. The exact regimen and timing are recorded in the eCRF, and sensitivity analyses will assess whether regimen selection influences feasibility or safety outcomes.

**Fig. 1:**
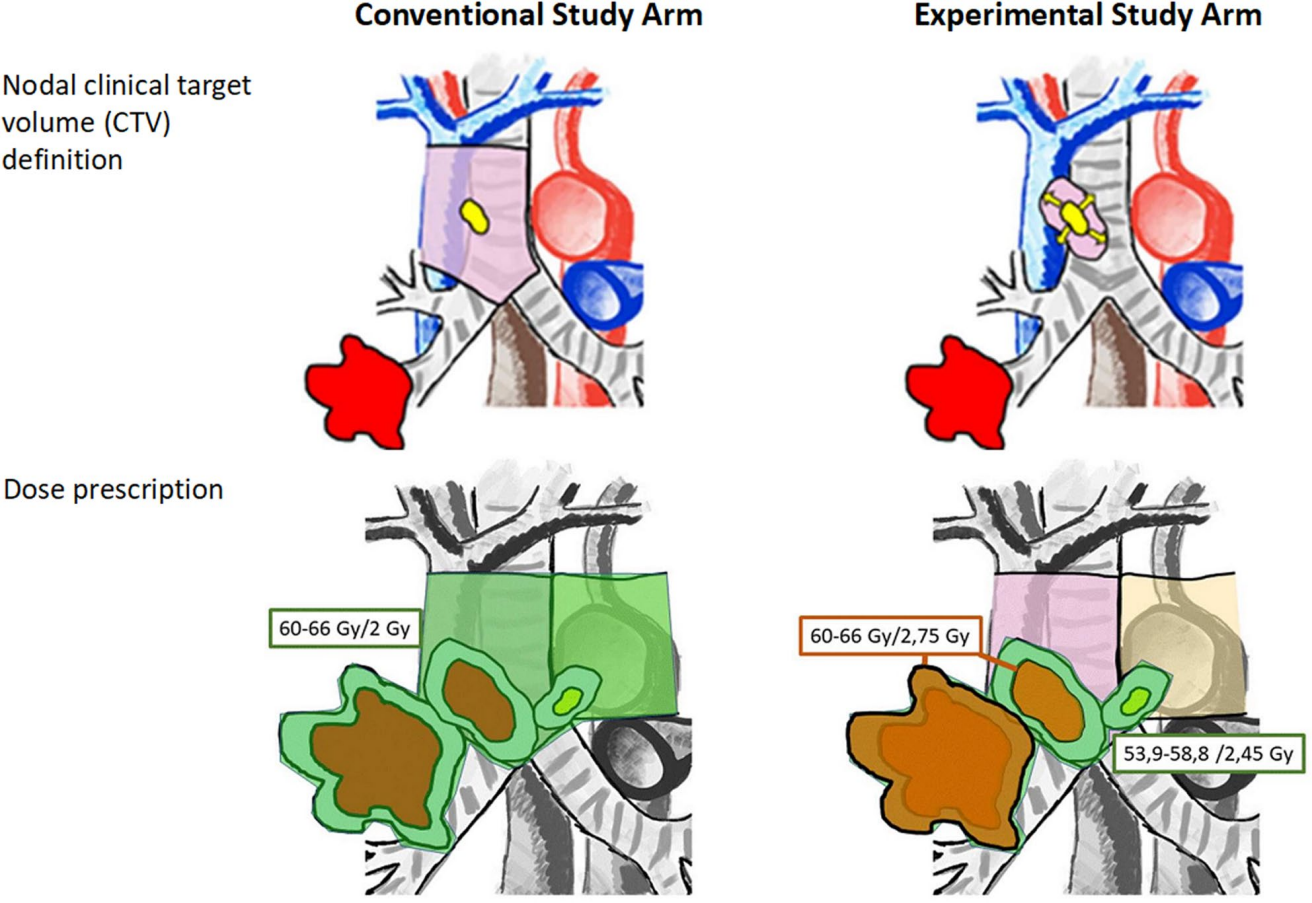
Nodal clinical target volume (CTV) definition and dose prescription inthe conventional and experimental study arm

Upon completion of CRT, patients in both arms who show at least stable disease according to the Response Evaluation Criteria in Solid Tumors (RECIST) v1.1, will be given durvalumab intravenously at a fixed dose of 1500 mg q4w. Durvalumab will be started within a maximum of 42 days after CRT completion provided all of the following predefined criteria are met (uniformly applied across arms): (i) no radiographic progression and at least stable disease (RECIST v1.1); (ii) ECOG 0–1; (iii) no pneumonitis ≥CTCAE grade 2 and no oxygen dependence; (iv) no systemic corticosteroids > 10 mg prednisone-equivalent/day or other chronic immunosuppression; (v) adequate organ function and no uncontrolled intercurrent illness including active infection. Reasons for non-initiation are prospectively documented in the eCRF. Durvalumab is administered as standard of care within the SmPC and continued for up to 12 months or until progression or unacceptable toxicity. The trial flowchart and study procedures are displayed in Fig. 1.

All patients are planned to stay in the study until the overall end of study. Scheduled efficacy, safety and survival follow-up (FU) visits will be done three-monthly for the first 24 months after initiation of durvalumab treatment and 6-monthly afterwards or until progression, whichever occurs first. Study and FU visits will encompass clinical examination including assessment of potential TRAEs and performance status, review of prior and concomitant medications, diagnostic chest and upper abdominal CT scan, QoL questionnaires and routine blood samples. All patients will be followed up for survival. For assessment of survival status at end of study, telephone contacts are allowed. A complete overview of scheduled assessments is displayed in Table 2. Most relevantly, concomitant treatment with other PD1/PD-L1 antibodies and with immunosuppressive medications is prohibited during the trial. A complete list of permitted and prohibited concomitant medications is detailed in the study protocol.

#### Assessment of efficacy and safety

For evaluation of tumor response, RECIST v1.1 criteria are used. For analysis of the exploratory objectives, patients with residual FDG uptake at end of CRT are planned to have an additional FDG-PET/CT assessment at the time of the primary endpoint (5 months visit; 5 months +/- 1 month after start of CRT). The decision to perform FDG-PET/CT assessment at the 5 months visit is primarily made based on PERCIST criteria, which are applied in addition to RECIST v1.1 criteria during tumor evaluation at the end of CRT [40, 41]. All observed adverse events (AEs) will be recorded in the eCRF and graded according to NCI-CTCAE v5.0 for all patients, and their relationship to all study treatments/procedures will be assessed and recorded. The decision to discontinue study treatment for individual patients on the basis of occurring toxicity will be made by the investigator. Detailed criteria for dose reduction or discontinuation of individual elements of the study treatment (radiotherapy, chemotherapy, immunotherapy) are listed in the trial protocol. QoL will be assessed according to the standardized questionnaires EORTC QLQ-C30 and the lung cancer-specific module QLQ-LC13. Questionnaires will be administered to the patients at the timepoints specified in the schedule of assessments (Table 2).

**Table 2:** Schedule of assessments (SPIRIT Diagram)

Procedure / Point in Time	Screening	*R*	RT Planning	CRTr		Immunotherapy (IO) and Follow Up									End of Study
	Inclusion			CRT		Immunotherapy					Safety and Survival Follow Up (FU)				
				Start of CRT	End of CRT	Start of IO	5 M visit^a^	8 M visit^a^	12 M visit^a^	End of IO	Safety FU 1	Safety FU 2	Long-term FU	End of Study	
Scheduling Window	d-21 - d0		prior to d1	d1	within 7d after end of CRT	max 42 days after end of CRT	5mo +/-1 after start of CRT	8mo +/-1 after start of CRT	12mo +/-1 after start of CRT	time of decision	30 + 7d after last dose of IO	30 + 7d after last dose of durvalumab	for timing see table caption^b^		
Informed Consent	x														
Inclusion/ Exclusion criteria	x														
Demographics (incl. tobacco use) and Medical															
History	x														
Prior and Concomitant Medication Review	x^c^			x	x	x	x	x	x	x	x^d^	x^d^			
ECOG performance status	x			x	x	x	x	x	x	x	x	x			
Physical Examination	x^e^			x^e^	As per clinical routine	x^e^	x^e^	As per clinical routine	As per clinical routine	As per clinical routine	As per clinical routine	As per clinical routine			
Vital Signs, O2 Satura- tion, Weight	x			x	x	x	x	x	x	x	x	x			
Pulmonary Function Tests^f^	x						x								
12-lead ECG and Echo- cardiogram	x			If clinically indicated	If clinically indicated	If clinically indicated	x	If clinically indicated	If clinically indicated	If clinically indicated	If clinically indicated	If clinically indicated	If clinically indicated		
Pregnancy Test – Urine or Serum ß-HCG^g^	x			x		x									
CBC with Differential^h^	x			x	x	x	x	x	x	x	x	x			
Comprehensive Serum Chemistry Panel^i^	x			x	x	x	x	x	x	x	x	x			
Urinalysis^j^	x			x	x	x				x	x				
TSH, FT3, FT4	x														
HBV, HCV diagnos- tics^k^	x														
MRI Brain (or CT Brain)	x														
Diagnostic Chest CT- Scan (incl. Upper Abdominal Scan)^l^	x				x		x	x	x	x^n^			x^n^		
FDG-PET/CT Scan	x				x		x^m^								
Planning CT	x^o^ (prior to start of RCT)														
RT Planning			x (prior to start of RCT)												
Upload of RT plan via eCRF for RT-QA				x											
QoL Questionnaires (EORTC QLQ C-30; QLQ-LC13)				x	x	x	x	x	x	x	x	x	x^b^		
Documentation of Radiotherapy				x	x										
Documentation of Chemotherapy				x	x										
Documentation of Immunotherapy						x	x	x	x	x					
Adverse Events^p^	◊			◊	◊	◊	◊	◊	◊	◊	◊	◊			
Documentation of Sub- sequent Therapy										x	x	x	x^b^		
Survival Status (if applicable date of death and reason for death)				x	x	x	x	x	x	x	x	x	x^p^	x^q^	
Reason for end of study and date of last contact														x	

*Abbreviations:* *R* Randomization, *CRT* Chemoradiotherapy, *d* Days, *m* Months, *QoL* Health-related quality of life, *IO* Durvalumab immunotherapy

^a^Visits should be performed for all patients who started durvalumab therapy and who are still in response or have stable disease at the time of respective visit, independently of the current status of durvalumab therapy

^b^Long term FU: All patients are planned to stay in study until overall end of study. For patients whose disease has not progressed at the start of long term follow up, the following assessments are planned for the first 24 months after start of durvalumab or until progression (whichever occurs first) every 3 months (+/- 6 weeks) within the second year from start of chemoradiotherapy and every 6 months +/- 3 months thereafter

Diagnostic Chest CT-Scan (incl. Upper Abdominal Scan)

QoL Questionnaires (EORTC QLQ C-30; QLQ-LC13)

Documentation of subsequent therapy

Survival status (if applicable date of death and reason for death) After that time, all patients alive should stay in the study until overall end of study

^c^Prior non-NSCLC-related medication used within the last 28 days prior to inclusion and non-NSCLC medication ongoing at study inclusion should be documented

^d^Only concomitant medication used for the treatment of adverse reactions should be documented

^e^full physical examination including general appearance, respiratory, cardiovascular, abdomen, skin, head and neck (including ears, eyes, nose and throat), lymph nodes, thyroid, musculosceletal (including spine and extremities), genital/rectal, and neurological systems and only at screening, height

^f^Pulmonary function tests include blood gas analysis + whole body plethysmography + diffusion capacity test and/or stress blood gas analysis [ideally both]

^g^Only for WOCBP. WOCBP should only be included after a negative highly sensitive urine or serum pregnancy test. If applicable, this test should be repeated a maximum of 24-hours before the first dose of chemoradiotherapy and before first dose of durvalumab therapy

^h^Hematology panel: Complete Blood Count with differential [white blood cell (WBC) count, absolute neutrophil count (ANC); absolute lymphocyte count (ALC); platelet count; hemoglobin]

^i^Chemistry panel: alkaline phosphatase; lipase; alanine aminotransferase (ALT); aspartate aminotransferase (AST); lactate dehydrogenase (LDH); calcium; glucose; potassium; sodium; total bilirubin; direct bilirubin (if total bilirubin is elevated above the upper limit of normal); blood urea nitrogen; C-reactive protein (CRP); gamma-GT, albumin, creatinine, calculated creatinine clearance (according to Cockcroft and Gault)

^j^Urinalysis by qualitative examination (stick) for: blood, glucose, proteins, nitrites, ketones, leucocytes, density, pH.

^k^Diagnostics include hepatitis B surface antigen (HBV sAg) and hepatitis C antibody (HCV Ab) or hepatitis C RNA (HCV RNA)

^l^If not done together with PET/CT

^m^Only for patients showing partial response or stable disease according to PERCIST criteria at the end of chemoradiotherapy. FDG-PET/CT scan should be performed irrespective of the durvalumab treatment status at this time. This means that also patients, who already discontinued from durvalumab or who didn´t start durvalumab will receive this FDG-PET/CT scan if they are still in the study and showed partial response or stable disease according to PERCIST criteria at the end of chemoradiotherapy

^n^If not already performed as part of clininical practice/ due to clinical indication within 4 weeks +/- date of decision

^o^Might also be performed after randomization but prior to the start of radiochemotherapy

^p^For documentation of adverse events please refer to section 10. Documentation should occur in an ongoing basis in line with the defined documentation timelines

^q^For patients not known to be deceased before overall end of study, survival status should be assessed at overall end of study. For modalities of assessment, please refer to section 8.1.4

^r^the first dose of chemotherapy is to be administered ± 7 days from the first dose of radiotherapy. The date of first administration of chemotherapy or radiotherapy is defined as day 1 of chemoradiotherapy

#### Assessment of exploratory parameters

To address PET-related exploratory objectives, several quantifiable PET-specific imaging parameters will be documented in the eCRF. Additionally, PET-based imaging data will be uploaded via the eCRF. Although the immunomarkers NLR and ALI will be assessed for this study, no specific biomarker sampling is required, as those parameters will be calculated from routinely performed laboratory assessments, as outlined in the schedule of assessments. The Effective Radiotherapy Dose to Immune Cells (EDIC) model considers the dose exposure of circulating immune cells. It will be calculated as described in the study by Xu et al., based on parameters extracted from the RT treatment plan [42]. 

### Radiotherapy

A mandatory diagnostic FDG-PET/CT scan is required within three weeks before the start of radiotherapy and shall be used for RT-planning. Planning in a mid-ventilation position or deep breath-hold with active breathing control devices is preferred. The PET/CT will be co-registered in the radiotherapy treatment planning system when acquired in the planning position, and the quality of co-registration will be checked before contouring.

**Fig. 2:**
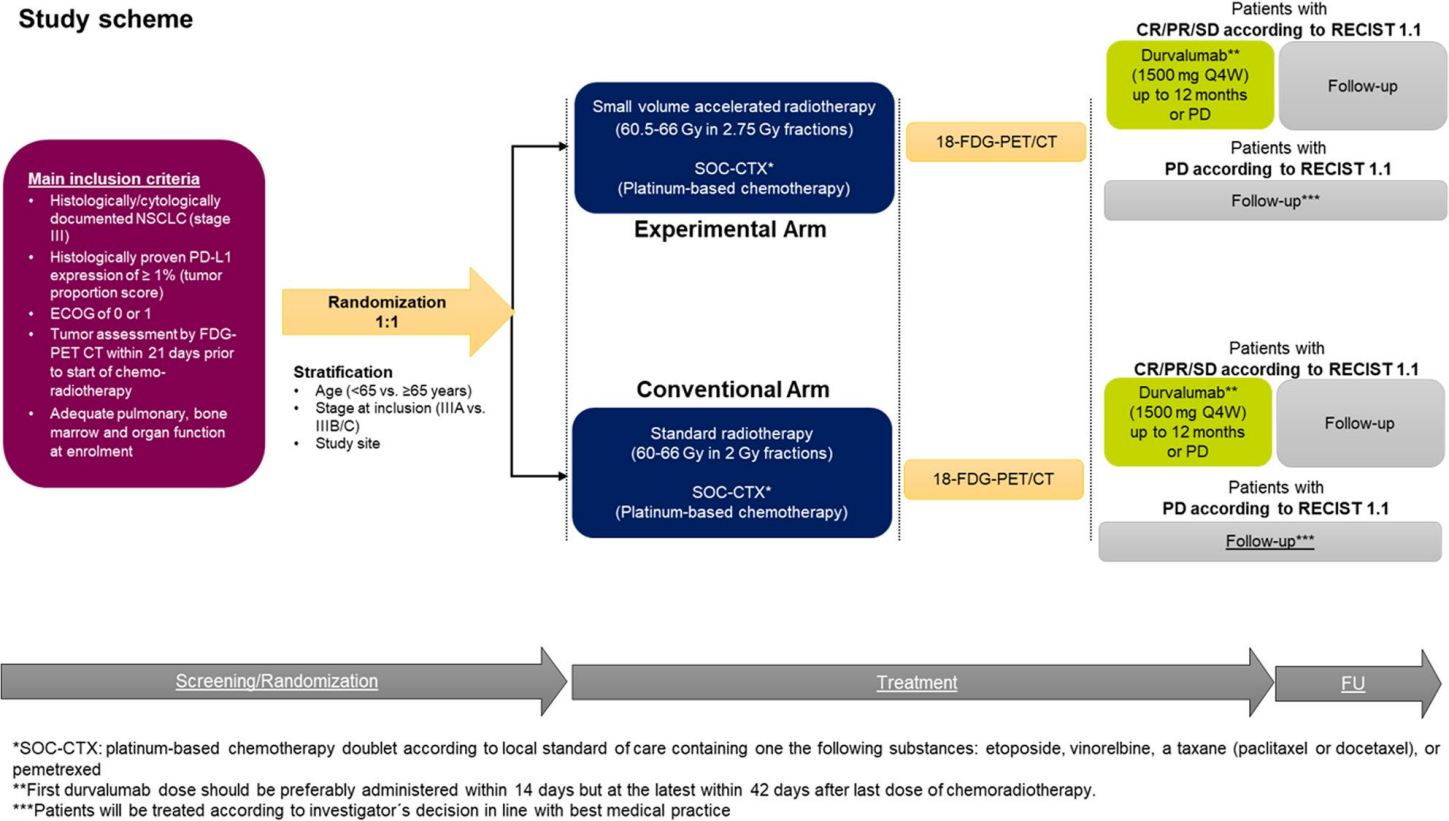
Flow Chart of the PACCELIO trial

The treating radiation oncologist will delineate the following target volumes (see Fig. 2):

- GTV delineation on the planning-CT is mandatory. The GTV of the primary tumor and lymph nodes will be contoured separately, if anatomically distinguishable. Delineation of GTV based on both CT and FDG-PET/CT is required. PET-based automatic contouring methods are permissible, if institutionally calibrated. PET-positive lymph nodes are included in the GTV. Lymph nodes that are enlarged on CT but not PET-positive will not be included in the GTV. PET-positive nodes may only be omitted if the FDG positivity can be clearly explained by a non-malignant biopsy or if a mediastinoscopy has been performed that did not reveal any malignancy in the lymph nodes. Lymph nodes that prove to be malignant through biopsy are delineated as GTV, even if they are not classified as pathological on PET. Bulky lymph nodes (≥3 cm in any diameter) are distinguished from non-bulky lymph nodes.
- CTV for the primary tumor is created by expanding the GTV to account for potential microscopic spread, with manual editing for surrounding anatomy. The primary tumor GTV will be expanded by 5-8 mm and adjusted manually to account for surrounding anatomy in both study arms. The nodal CTV definition, based on the two options of the ESTRO ACROP guideline, is subject to randomization: In the Experimental Arm, nodal GTV will be expanded by 5-8 mm and adjusted for surrounding anatomy (ACROP option 2) [23]. In the Conventional Arm, nodal CTV will include the whole pathologically affected lymph node level according to the Mountain-Dresler definitions (ACROP option 1) [43].
- A PTV margin of 6-10 mm will be added according to institutional standard of care to account for technical and positioning uncertainties.

Organs at risk to be accounted for in treatment planning include separate and combined lungs (excluding GTV), the entire heart (and cardiac sub-structures), the esophagus, and the spinal canal. Additional structures such as the brachial plexus may be contoured if in the vicinity of the target. Dose constraints to normal lung tissue, spinal cord, esophagus, and heart are applied rigorously and according to pre-specified RTQA requirements.

The prescribed dose is randomized between the conventional and Experimental Arms, as specified under 2.2.2, and shown in Fig. 2.

In both arms, intensity modulated radiotherapy (IMRT) will be employed, adhering to International Commission on Radiation Units and Measurements (ICRU) 83 standards [44]. During treatment, regular patient positioning verification and isocenter localization will be performed radiologically using cone/fan-beam CTs or conventional X-rays.

### Radiotherapy Quality Assurance (RTQA)

To ensure protocol-consistent and uniform RT across centers, sites must pass a pre-activation dummy run: a sample case is planned per protocol and undergoes central quality control prior to first inclusion. For each treated patient, pseudonymized DICOM-RT plans together with the planning FDG-PET/CT are uploaded via the eCRF at treatment onset or within 3 weeks of RT day 1 for centralized review. Planning and delivery follow ICRU 83 with IMRT (volumetric techniques preferred), arm-specific prescriptions, and predefined OAR constraints listed in an RTQA manual, which also contains detailed contouring, dosimetric specifications, and definitions of major and minor protocol deviations.

### Study endpoints

#### Primary outcome

The primary endpoint of the PACCELIO trial is treatment feasibility, defined as the rate of patients completing study treatment in the Experimental Arm, compared to the Conventional Arm. Treatment completion is defined as (i) receiving the prescribed RT dose ± 2 fractions, and (ii) simultaneous platinum-based chemotherapy and (iii) immunotherapy consolidation with durvalumab starting within 42 days after the last dose of chemoradiotherapy, and (iv) at least 3 doses of durvalumab. Patients who received fewer than three doses of durvalumab and subsequently experienced extrathoracic, immune-related adverse events necessitating permanent discontinuation of immunotherapy will be considered to have completed the treatment protocol.

##### Rationale 

The endpoint is designed to capture the component of treatment completion most relevantly influenced by radiotherapy fractionation, namely, completing CRT and early transition into consolidation. Requiring the full 12-month durvalumab course would introduce substantial confounding from late immune-related toxicities and patient-level factors unrelated to RT fractionation. A threshold of ≥ 3 q4-weekly doses (~ 12 weeks) pragmatically confirms both initiation and maintenance through the early consolidation window when failures to start and early discontinuations are most likely to occur and most closely linked to CRT tolerance and recovery. This approach aligns with the PACIFIC standard (durvalumab up to 12 months, typically initiated within 42 days of CRT) and with observational data associating early discontinuation ( < ~ 2 months) with inferior outcomes and indicating that symptomatic pneumonitis clusters within 1–6 months post-CRT [12, 45–47]. 

#### Secondary outcomes

Secondary efficacy endpoints include time to local and locoregional, as well as distant progression, progression-free survival (PFS), overall survival (OS), objective response rate (ORR) and disease control rate (DCR). Safety endpoints are assessed according to CTCAE v5.0. Quality of Life is assessed according to EORTC QLQ-C30 and QLQ-LC13 questionnaires. Exploratory outcomes include assessment of Radiotherapy Quality Assurance (RTQA), the effect of quantifiable FDG-PET/CT imaging parameters, as well as prognostic immunomarkers (NLR and ALI) on outcome measures.

#### Retention

The PACCELIO trial includes plans to promote participant retention and complete follow-up by ensuring continued monitoring, data collection, documentation of subsequent therapies, and collection of adverse events for participants who discontinue or deviate from intervention protocols, including the assessment of survival status through up to three telephone contacts.

#### Monitoring and regulatory compliance

This study will be performed in compliance with the Declaration of Helsinki principles, and the protocol was accepted by the responsible institutional review boards, as need to be all future protocol modifications. Protocol amendments will be immediately communicated to investigators, trial participants, regulators and trial registries, as required by ICH-GCP. Compliance with the protocol, Standard Operating Procedures (SOPs), and ICH-GCP guidelines, specifically for reporting managing adverse events, will be ensured through regular quality audits by the sponsor, with investigators required to support audits and inspections, providing direct access to source data. Study site personnel will receive training before study initiation, and ongoing monitoring visits will verify patient rights, data accuracy, and protocol compliance, with all issues addressed promptly to maintain data quality and regulatory adherence. Frequency and scope of the monitoring visits are defined in the monitoring plan. An independent Data Safety Monitoring Board (DSMB) composed of specialists (biometrician, radiation oncologist, oncologist, pulmonologist) will regularly review study progress and safety events, making recommendations on study continuation, modification, or termination by the sponsor, based on safety concerns or if new data contradictory to the study setup emerge. In accordance with local law, ICH-GCP and General Data Protection Regulation (GDPR), patient confidentiality will be protected by ensuring pseudonomized data collection, transmission and evaluation. In accordance with local law, all study patients are insured up to a sum of 500.000 EUR.

#### Publication of trial results

The Principal Investigator will have unlimited access to the pseudonymized final trial dataset. Following final analysis, trial results will be published in a peer-reviewed journal, adhering to the International Committee of Medical Journal Editors (ICMJE) authorship guidelines and irrespective of trial outcome. With final publication of the results, the full protocol will be made available publicly.

### Statistical analysis

Statistical analysis adheres to the ICH Guidelines for clinical study reports and trials, utilizing applicable SOPs.

#### Sample size calculation

The hypothesis is that FDG-PET-based reduced-volume, hypofractionated, accelerated CRT followed by durvalumab (Experimental Arm) will result in a higher treatment completion rate (as defined above) compared to FDG-PET-based standard-volume, conventionally fractionated CRT followed by durvalumab (Conventional Arm). Randomization is in a 1:1 ratio, stratified by age, stage at inclusion, and study site. To detect an improvement in treatment completion rate from 60% to 85% with a 5% alpha and 80% power, 49 patients per arm are required, totaling 110 patients when factoring in a 10% dropout rate. The 60% control-arm estimate was chosen to reflect contemporary real-world transition rates to durvalumab after CRT, which have increased from early post-PACIFIC reports (~ 50%) to ~ 60–68% across multiple cohorts (2018–2022); thus, 60% represents a conservative, externally grounded baseline for current standard of care. Feasibility of adequate participant enrolment has been determined by analysing the yearly treated number of cases matching the inclusion criteria for the participating sites. With 11 planned sites, the required average recruitment is 5 patients per site per year. All participating sites have the required expertise and infrastructure, as well as past experience of recruiting NSCLC patients into clinical trials.

#### Data management

Data is entered using an electronic data capture (EDC) system containing the eCRF, ensuring pseudonymized data entry, audit trails, automated and manual checks for data quality, secure data storage and transmission, and compliance with GCP, FDA, and EU regulations, with detailed procedures outlined in the Data Validation Plan and Data Management Plan.

#### Methods of statistical analysis

The Intention-to-Treat (ITT) population includes all randomized patients for primary and secondary efficacy evaluations, the Per-Protocol (PP) population includes ITT patients meeting all criteria without major protocol deviations for secondary efficacy analyses, the Quality of Life Set includes patients with baseline and post-baseline QoL scores analyzed based on ITT and PP populations; and the Safety Population includes all patients receiving at least one study treatment for safety endpoints analysis. The primary endpoint of treatment completion (as defined above), will be analyzed on the ITT and PP populations using Fisher’s Exact test, comparing the Experimental and Conventional Arms. Safety endpoints will be analyzed using frequency tables, detailing severity grade per NCI-CTCAE 5.0, with listings for all Serious Adverse Events (SAEs). Efficacy outcomes will be analyzed for ITT and PP populations, employing Kaplan-Meier techniques and a competing risk framework for specific endpoints. Patient-reported health-related quality of life analysis will follow respective manuals. Exploratory objectives will be analyzed separately, with results provided in distinct reports. The primary analysis is scheduled approximately 12 months after the last patient’s initiation of durvalumab therapy, covering primary and secondary endpoints (except OS and QoL). Analysis results will be updated subsequently following database closure. Further details on statistical analysis are documented in the study protocol, as well as as the statistical analysis plan (SAP), drafted prior to trial initiation and finalized during a blinded data review.

### Trial status

As of July 2024, 7 sites (6 in Germany, 1 in Switzerland) are initiated. The first patient was enrolled on 15 July 2024. The PACCELIO trial is currently recruiting patients.

## Discussion

A central challenge in managing unresectable stage III NSCLC, particularly during and post-CRT, involves ensuring patient compliance and achieving intrathoracic tumor control while preventing severe toxicity. These elements are crucial in enabling patients to proceed to consolidation immunotherapy, a critical component of the multimodality treatment to maximize disease control and survival. The PACCELIO trial addresses this unmet clinical need by combining FDG-PET/CT-based target volume reduction, hypofractionated accelerated dose delivery, and consolidation durvalumab immunotherapy, reflecting the latest pivotal advancements in stage III NSCLC treatment.

The use of FDG-PET/CT for precise radiotherapy planning allows for a reduction of radiation exposure to sensitive organs at risk, such as the heart, lungs and esophagus [21]. It also reduces the dose delivered to immune cells, thus potentially enhancing the response to consecutive immunotherapy, as supported by recent studies [48, 49]. This aligns with the findings of the PET-Plan trial, which supports a more targeted and volume-reduced approach in radiotherapy for unresectable stage III NSCLC. By focusing on biologically active areas of disease, FDG-PET/CT allows for more accurate target volume definition, thereby reducing radiation exposure of adjacent organs at risk and associated toxicities, while maintaining or even improving tumor control [20, 21]. 

The combination of target volume reduction with hypofractionated, accelerated dose delivery may deliver synergistic effects on outcome. While conventional fractionation is currently the standard, the potential benefits of hypofractionation include a condensed treatment schedule and potentially increased biologically effective dose [31, 32]. Notably, in other cancers such as breast cancer, where moderate hypofractionation has been more extensively studied, a reduction in acute and late toxicity has been observed with hypofractionated regimens [50]. Emerging evidence suggests that shorter treatment durations may enhance patient compliance and increase the likelihood of advancing to immunotherapy, addressing a critical treatment challenge [11, 35]. Additionally, the socio-economic benefits of reducing the overall treatment duration cannot be overlooked, as they may contribute to improved patient quality of life, reduce financial burden, and promote responsible healthcare resource utilization [51, 52]. 

The trial’s translational and exploratory outcomes include the prospective, longitudinal evaluation of immunomarkers like NLR and ALI, as well as immune-related radiotherapy planning parameters such as the EDIC and PET-related imaging parameters [18, 19, 42, 53, 54]. These analyses will contribute to the growing understanding of immune-related effects in NSCLC and their impact on cancer progression and response to multimodal treatments.

In summary, the PACCELIO trial will contribute valuable insights into the efficacy and feasibility of combining volume-reduced, hypofractionated, accelerated CRT with consolidative immunotherapy, potentially improving outcomes of patients with unresectable stage III NSCLC. Patient recruitment for the PACCELIO trial started in June 2024, and participating facilities across Germany, Switzerland and Austria are currently enrolling patients who match the eligibility criteria.

## Roles and responsibilities

### Coordinating centre

• Prof. Dr. Nestle (Department of Radiation Oncology, Kliniken Maria Hilf, Moenchengladbach, Germany) and Prof. Dr. Rieken (Department of Radiation Oncology, University Hospital Göttingen, Göttingen, Germany):
  - ◦ National Coordinating Investigators
  - ◦ Medical Experts
  - ◦ Responsible for Medical Study Oversight

### Data and Safety Monitoring Board (DSMB)

- Dr. Jochem König (University Medical Center, Mainz, Germany), Biometrician.
- Prof. Thomas Brunner (Department of Radiation Oncology, Medical University of Graz, Graz, Austria), Radiation Oncologist.
- Prof. Martin Reck (Department of Thoracic Oncology, Lung Clinic Grosshansdorf, Großhansdorf, Germany), Oncologist.
- Prof. Rudolf Maria Huber (Department of Pulmonology, University Hospital of Munich, LMU, Munich, Germany), Pulmonologist.

The DSMB will provide the sponsor and study management with advice and support regarding patient safety in this clinical trial.

### Data management

• Magdalena Hofmann (Clinical Operations – Medical Writing - Alcedis GmbH, Gießen, Germany):
  - ◦ Responsible for Quality Assurance of entered data into the electronic Case Report Form (eCRF)

### Quality Assurance (QA)

• Radiotherapy QA:
  - ◦ Dr. Schimek-Jasch (Department of Radiology, Medical Center, Faculty of Medicine, University of Freiburg, Freiburg, Germany):
    - ▪ Quality Assurance regarding radiotherapy procedures

• PET/CT QA:
  - ◦ Prof. Dr. Miederer (Department of Translational Imaging in Oncology, National Center for Tumor Diseases (NCT/UCC) Dresden, University of Technology Dresden, Germany):
    - ▪ Quality Assurance for PET/CT imaging

## Supplementary Information

Supplementary Material 1.

## Data Availability

No datasets were generated or analysed during the current study.
